# Revealing the self in a digital world: a systematic review of adolescent online and offline self-disclosure

**DOI:** 10.1016/j.copsyc.2022.101309

**Published:** 2022-03-21

**Authors:** Emily Towner, Jennifer Grint, Tally Levy, Sarah-Jayne Blakemore, Livia Tomova

**Affiliations:** 1 Department of Psychology, University of Cambridge, UK; 2 Institute of Cognitive Neuroscience, University College London, UK; 3 Hughes Hall, University of Cambridge, UK

**Keywords:** self-disclosure, adolescence, social needs, digital communication, systematic review

## Abstract

Adolescence is an important stage of social development. While adolescents are prominent adopters of social media, little is known about whether digital interactions can fulfil the social needs of this age group. Here, we focus on one component of social interaction: self-disclosure. In a systematic review, we investigate the role of self-disclosure in adolescent relationships and the differences between online and offline self-disclosure. The results suggest that self-disclosure is associated with higher relationship quality and well-being. Online self-disclosure appears to be less fulfilling and beneficial for relationship quality than face-to-face self-disclosure. However, certain populations appear to benefit more from online than offline self-disclosure - such as highly anxious adolescents and boys aged 12-13 years, who prefer to first self-disclose online before engaging in offline self-disclosure. This suggests that both online and offline self-disclosure can play a role in fulfilling adolescent social needs.

## Introduction

Social interaction is a basic human need^1,2^. Adolescents (10-24 years^3^) have a particularly high need for social interaction with peers^4^. Technological advances in the past decade or so have radically changed the amount and types of social interaction young people experience. 83% of young people aged 12–15 years in the UK own a smartphone and, between 2015 and 2019, approximately 70% had a social media profile^5^. As social interactions are increasingly moving into the virtual domain, it is important to understand whether and how technology is enabling young people to fulfill their social needs.

‘Self-disclosure,’ in which an individual reveals personal information to another^6,7^, is an important building block for relationships^8^. Self-disclosure is thought to help form and maintain social relationships as it builds a bond of trust between individuals^9^. An early meta-analysis suggested that people who self-disclose tend to be more liked, and that people like those to whom they self-disclose^10^. In addition, it has been proposed that self-disclosure is inherently rewarding in that people are willing to forgo money in order to disclose information about themselves^11,12^.

Modern advances in technology have changed the way in which people communicate. Some studies suggest that the ability to engage in selective self-presentation might increase self-disclosure^13^. Others propose the lack of non-verbal cues in computer-mediated communication may lead to decreased self-disclosure^14^. Similarly, while self-disclosure online can help to build trust within a relationship^15,16^, it has been proposed that self-disclosure conducted via computer-mediated communication is less fulfilling than face-to-face self-disclosure^17^.

Here, we conducted a systematic review to investigate two research questions: Q1: How is self-disclosure important for relationship-building in adolescence?; and Q2: Is this different for online and offline communication? The primary aim of our systematic review was to ascertain the ways in which self-disclosure is important for building social relationships, and thus can fulfil social needs, in adolescence. This involved identifying the appropriate recent literature and identifying key themes relating to factors that affect self-disclosure and the impact it has on social relationships. From there, we sought to compare the efficacy of online communication and offline interaction in the fulfilment of these social needs. Online communication offers fewer nonverbal cues (such as facial expressions in text-based messaging) and higher anonymity^18^ and therefore differs from face-to-face interactions in substantial ways. Previous research in adults has shown mixed findings regarding the amount of self-disclosure in online versus offline contexts^19^. Given that adolescents spend more time than adults socializing via digital means^20^, we hypothesized that self-disclosure in social interactions in adolescence would be affected differently by online communication versus offline communication.

## Methods

Following the PRISMA guidelines^21^, we conducted a systematic literature review to answer our two separate, but related, research questions into self-disclosure and adolescent social needs:

Q1: How is self-disclosure important for relationship-building in adolescence?

Q2: Is this different for online and offline communication?

The review was preregistered in the Open Science Framework database at https://osf.io/pf5ty (OSF, 2021).

### Search strategy and inclusion criteria

We searched the SCOPUS database for papers published between 2010 and 2021, which were related to the use of self-disclosure in adolescence in both offline and online communication.

To achieve this, we used the following search term:

(TITLE-ABS-KEY (((adolescen* OR teen*) AND (“self-disclosure” OR “informing others” OR “social sharing”) AND (virtual OR “computer-mediated” OR online)) OR ((adolescen* OR teen*) AND (“self-disclosure” OR “informing others” OR “social sharing”))) AND “social” AND (PUBYEAR > 2009)) AND NOT (“clinical”)

Our inclusion criteria meant that, whether the study was conducted online or offline, only papers that studied self-disclosure in a social context among 10-24-year-olds were included in the final review. Our exclusion criteria meant that papers published before 2010 were excluded, to ensure we were discussing recent findings that potentially involved online communication. We also excluded papers that discussed self-disclosure in a clinical or familial setting, or that assessed self-disclosure as a personality trait. Finally, we excluded reviews and empirical papers that included qualitative data only.

### Data extraction and analysis

Two independent reviewers searched and screened the literature. 218 studies were retrieved from the SCOPUS database. We determined that 38 of these studies were potentially relevant and needed to be read in full to test their eligibility. Overall, 19 studies were included in the final review. The exclusion criteria, as described above, were used to select the papers that would be included for the systematic literature review. Papers were thus excluded for: (1) having an incompatible age range of participants (i.e., outside of 10-24 years); (2) being focused on relationships other than those among peers; (3) studying personality traits rather than social relationships; (4) being a review rather than an empirical study; or (5) having a qualitative design. This left 19 papers to be included.

Of the 19 studies, 5 (26%) included samples from the USA, a further 8 studies (42%) included European samples (based in the Netherlands, Belgium, Germany, Sweden, or Turkey), and the final 6 studies (32%) were conducted in Asian countries (Taiwan, Malaysia, China and Singapore). The age range of participants across studies was 10–22 years and all studies were quantitative by design and used self-report measures of self-disclosure. For the majority of studies (7 or 37%), the authors had developed their own measure of self-disclosure; this was more common among the studies that were measuring online self-disclosure, where established measures are yet to be developed.

### Methodological quality assessment

Methodological and reporting quality of each record included in the systematic review was assessed using an adapted version of the Downs and Black Checklist^22^. The original checklist was adapted to suit the non-interventional nature of the research question. Two reviewers (ET and TL) critically appraised each study and sum scores were converted into percentages. Notes regarding critical appraisal were kept and major disagreements between co-reviewers were resolved by a third reviewer (LT). After resolving major disagreements, interrater reliability was assessed. The intraclass correlation coefficient (ICC) estimate and its 95% confidence interval was calculated using R package irr^23^, based on a mean-rating (k = 2), consistency, 2-way mixed-effects model. The ICC was 0.76 with a 95% confidence interval from 0.37 to 0.91 (*F*(18,18) = 4.09, *p* = .002), indicating good reliability. For each study, the two reviewer scores were averaged; mean quality assessment scores are reported in Table 1. The adapted checklist, quality assessments, and other supplementary materials can be found on the OSF (https://osf.io/pf5ty).

## Results

### Self-disclosure and relationship-building in adolescence

Twelve (63%) of the studies included in the review explored the association between relationship quality and adolescent self-disclosure. All of these studies proposed that adolescent self-disclosure improves relationship quality as it builds trust between peers^24–36^. For adolescents, self-disclosure indicates a commitment to the peer and to the relationship with the peer^31^, strengthening it and leading to more reciprocal friendships^34^. Two papers expanded upon this, suggesting that self-disclosure improves relationship quality because it creates a way to build social support^32,37^. Overall, our review suggests that self-disclosure improves relationship quality among adolescents.

Furthermore, all reviewed papers highlight a positive relationship between psychological well-being and adolescent self-disclosure. Two papers^38,39^ showed that adolescent self-disclosure predicts psychological needs satisfaction, and, in turn, life satisfaction. One paper suggested this may be because adolescents find self-disclosure to be a rewarding activity^35^, and so engaging in it has positive effects for wellbeing (especially for males^29^). Other possible explanations for the link between wellbeing and self-disclosure include the notion that it allows individuals to explore and form their own identity^40^, a key part of adolescent development^41^. A bidirectional relationship was identified here, suggesting that those with higher psychological wellbeing engage in more self-disclosure^34^.

### Differences between online and offline self-disclosure

Of the papers included in the final review, 9 (48%) of them investigated online adolescent self-disclosure, 5 (26%) investigated offline communication, and the final 5 (26%) compared the two. Our findings suggested that, while the frequency of both online and offline self-disclosures was linked to better relationship quality^32,33,37^, adolescents showed a preference for face-to-face communication^25^. Nevertheless, there was evidence that online self-disclosure is a useful tool for bridging social capital, especially during significant life transitions^32^) and for practicing the social skills required for face-to-face communication^26,27,42,43^.

More specifically, three studies found that online self-disclosure provides unique benefits for the fulfilment of adolescents’ social needs. Koutamanis et al (2013) looked at how adolescents’ online social behaviour influences their ability to initiate offline friendships. This study used a longitudinal design measuring the use of instant messaging, participants’ ability to initiate offline friendships, diversity of the groups of people who participants communicated with online and online self-disclosure behaviour. Instant messaging was positively associated with participants’ ability to initiate offline friendships. This was due to the increased diversity of the people they were able to communicate with online positively influencing the initiation of offline friendships. Courtois and All (2012) looked at how learning information from another person’s online profile affects in-person relationships and communication by using questionnaires on online uncertainty reduction, level of certainty, self-disclosure and social anxiety. They found that particularly for individuals who scored high on a social anxiety scale (Social Interaction Anxiety Scale; SIAS^44^) looking at online profiles can provide information about what people are like offline, which can alleviate social anxiety. Valkenburg et al. (2011) used a longitudinal design to assess intimate online and offline self-disclosure using questionnaire measures. All participants used online self-disclosure to improve offline communication skills, demonstrating how online self-disclosure influences offline self-disclosure.

### Exploring moderating factors: gender differences and type of self-disclosure

We also explored whether the literature identifies moderating factors in the effects of self-disclosure such as gender and type of disclosure. Overall, five studies found that female adolescents self-disclosed more than male adolescents^24,29,31,34,40^. Female adolescents may have been reported as self-disclosing more as they begin doing so from a younger age and their friendship networks were defined by self-disclosure and commitment to their peers, unlike male friendship networks^29^. In contrast to this, two papers found higher self-disclosure in males than in females^32,45^ while three papers did not find significant gender differences^27,39,43^. One study reported a gender difference in text-based self-disclosure, but did not report whether disclosure was higher in males or females^46^. Interestingly, one longitudinal study found that particularly young boys (12-13-year-olds) preferred online compared to offline self-disclosure and that this effect disappeared after age 13 when boys showed a sharp increase in offline self-disclosure^47^. In addition, two studies found that boys disclose more demographic (e.g., birth date, hometown) and contact information online than girls^32,34^.

While the observed effects of self-disclosure (such as an association with higher relationship quality) were found across different measures of self-disclosure (see Table 1 for details), few studies directly compared effects of different types of self-disclosure outside of comparing online vs. offline self-disclosure. One study compared the effects of superficial to intimate self-disclosure in adolescent girls and found that girls preferred to self-disclose superficial information about themselves rather than intimate information – an effect that was higher in girls with high self-esteem^35^. The authors found that while sharing intimate information was undertaken with caution by all participants, particularly girls with high self-esteem showed a strong preference to share superficial information about themselves.

Almost none of the reviewed studies reported any negative effects of self-disclosure except for one^33^ that assessed regret about online self-disclosure. The authors found that particularly teens who use social network sites (SNS) more frequently and have more SNS friends (particularly unfamiliar friends) report regret about online postings more often.

In sum, our systematic review supported our first hypothesis by showing that self-disclosure is an important tool for building relationships in adolescents. Furthermore, our results also supported our second hypothesis that online and offline self-disclosure have different effects by showing that online self-disclosure was less fulfilling compared to offline self-disclosure. However, certain populations, such as highly anxious young people and young boys aged 12-13 years, appear to benefit more from online self-disclosure than offline self-disclosure.

## Discussion

Our first research question asked how self-disclosure affects relationship-building in adolescence. Our systematic review showed that self-disclosure is an important tool for building trust and showing commitment, as well as improving psychological wellbeing in young people aged 10-22 years. Our second research question asked how self-disclosure differs in online compared to face-to-face social interactions. A recent review has identified core features of online social interactions that distinguish them from face-to-face communication^18^: They provide fewer nonverbal cues (such as facial expressions in text-based messaging), higher anonymity, more opportunity to form new social ties across geographical distances and wider possibilities of dissemination of information (i.e., a social media post can reach thousands of people in a short amount of time, which is difficult in face-to-face interactions). Thus, online social interactions differ from face-to-face interactions in substantial ways. While they offer opportunities for socializing that are not possible in face-to-face interactions (such as interactions across geographical distances and reaching a high number of people in short time) they also represent a more limited way of interacting with others (by lacking nonverbal cues and having higher anonymity).

Online self-disclosure appears to be less fulfilling and beneficial for relationship quality than face-to-face self-disclosure. However, the results of our systematic review also indicated that online self-disclosure can be beneficial for highly anxious young people as it allows them to seek out more information about their peers, thus reducing their uncertainty around a relationship. More specifically, adolescents with higher levels of anxiety were shown to engage more in gathering information about friends on social networking sites (for example, by viewing their online profiles), which in turn mediated the negative relationship between social anxiety and levels of certainty about a friendship. This indicates that online self-disclosure can be helpful for young people with high social anxiety.

Our review also showed that adolescent self-disclosure is affected by gender. Despite inconsistent results of the impact that gender may have on adolescent self-disclosure, several studies suggested that female adolescents engage in self-disclosure more than male adolescents. Previous research suggested that this gender difference in self-disclosure occurs, partly because of differences in the assumed gender stereotypes in society^48^. It is often assumed that females engage in more self-disclosure than males because they are more ‘socially oriented^49^’ while the ideal of male toughness and stoicism may be able to predict difficulties in self-disclosing (especially personal information^50^). Societal gender stereotypes might explain gender differences in adolescent social behaviour as adolescents are only starting to form their own identity, with their actions largely guided by what they have seen in wider society already. Surprisingly, some studies showed that the gender difference observed for offline self-disclosure are reversed in the online world – here males tend to disclose more, particularly when it comes to demographic and contact information. In line with this, a longitudinal study showed that young boys aged 12-13 years preferred online self-disclosure compared to face-to-face self-disclosure which then stimulated offline self-disclosure after the age of 13 in these boys^47^. The authors found support for a “rehearsal hypothesis” in their data meaning that adolescents use the Internet as a relatively safe place where they can rehearse their self-disclosure and self-presentation skills (e.g.,^51,52^). This finding is intriguing as it suggests a unique role of online social interactions in the development of social skills for young boys.

Overall, our results support Altman and Taylor’s (1973) Social Penetration Theory^9^, which provides an explanation for the role of self-disclosure in the formation and maintenance of interpersonal relationships. Altman and Taylor proposed that social relationships develop in quality as self-disclosure brings breadth and depth to the relationship and increases trust between the individuals.

An alternative explanation is provided by uncertainty reduction theory^53^, which suggests that people self-disclose and seek disclosure from others to reduce uncertainty at any point in their relationships. Such an account also considers the reciprocal nature of relationships and might explain the finding from our systematic review that online self-disclosure was overall seen as less fulfilling than face-to-face self-disclosure. There is, in general, more uncertainty during online self-disclosure due to the added anonymity and lack of nonverbal cues in online communications, and therefore self-disclosure during online interactions might be less successful in reducing uncertainty in relationships. However, for young people who find social interactions threatening or have less experience with them, face-to-face self-disclosure might not carry these beneficial features, while online self-disclosure might provide a safer way to engage with others. This in turn might help certain young people (such as people who are very anxious and young boys aged 12-13 years) to strengthen their face-to-face relationships.

## Conclusion

Our results suggested that self-disclosure is important in fulfilling adolescent social needs as it improves interpersonal relationship quality (by building trust) and individual psychological well-being. Our review also showed that online self-disclosure is less successful in fulfilling adolescent social needs although certain populations (such as young people with high anxiety and young boys) might benefit more from online-self disclosure.

Our findings are relevant in light of the social distancing measures in response to the COVID19 pandemic that have prevented in-person socializing. Many young people were compelled to socialize with peers mainly -or exclusively- via social media. Indeed, recent studies^54^ reported that 13.8% of adolescents (aged 17-22 years) often or always felt lonely as a result of lockdowns and other restrictions on socializing. Thus, online social interactions might provide unique ways to deal with such restrictions on face-to-face social interactions and help promote feelings of connectedness in young people.

## Future Research Directions

Our systematic review has highlighted the role of self-disclosure for satisfying social needs of adolescents and has shown that online self-disclosure can complement face-to-face self-disclosure. However, it is only a first step in evaluating how components of social interactions differ in in-person versus in computer mediated settings. Future research should assess self-disclosure in boys and girls early in adolescence and follow their development into their late teens to understand the role of (online and offline) self-disclosure in the development of social skills.

Furthermore, our systematic review revealed that only few studies compared different types of self-disclosure outside the comparison of online vs. offline self-disclosure. Only one study compared intimate vs. superficial self-disclosure in adolescent girls and found that superficial self-disclosure might have a unique role in adolescent social interactions as it was experienced as rewarding and adolescents preferred to engage in it more compared to intimate self-disclosure which carries more risk of rejection. Future studies should compare the role of intimate vs. superficial self-disclosure in more detail and also assess the effects of gender and age. For example, does this preference change across adolescent development?

Our systematic review showed only one study that assessed negative effects of self-disclosure which was measured as regret of posting information about oneself. This study found that particularly young people who use social media often and have large (anonymous) friend groups on social media tend to experience regret more often. More research is needed on the potentially negative effects of disclosing information about oneself online – and how these effects affect mental well-being of young people.

Finally, as social interactions and relationships with peers are undergoing massive changes during adolescence, it will be invaluable to conduct more future studies using longitudinal designs. These longitudinal designs will also allow the investigation of gender differences, and other mediating factors in more detail. Ultimately, understanding how adolescents use social media to build relationships with peers can also provide the basis for developing effective recommendations for parents on whether and how they should limit/monitor social media use of their children.

## Figures and Tables

**Figure 1:**
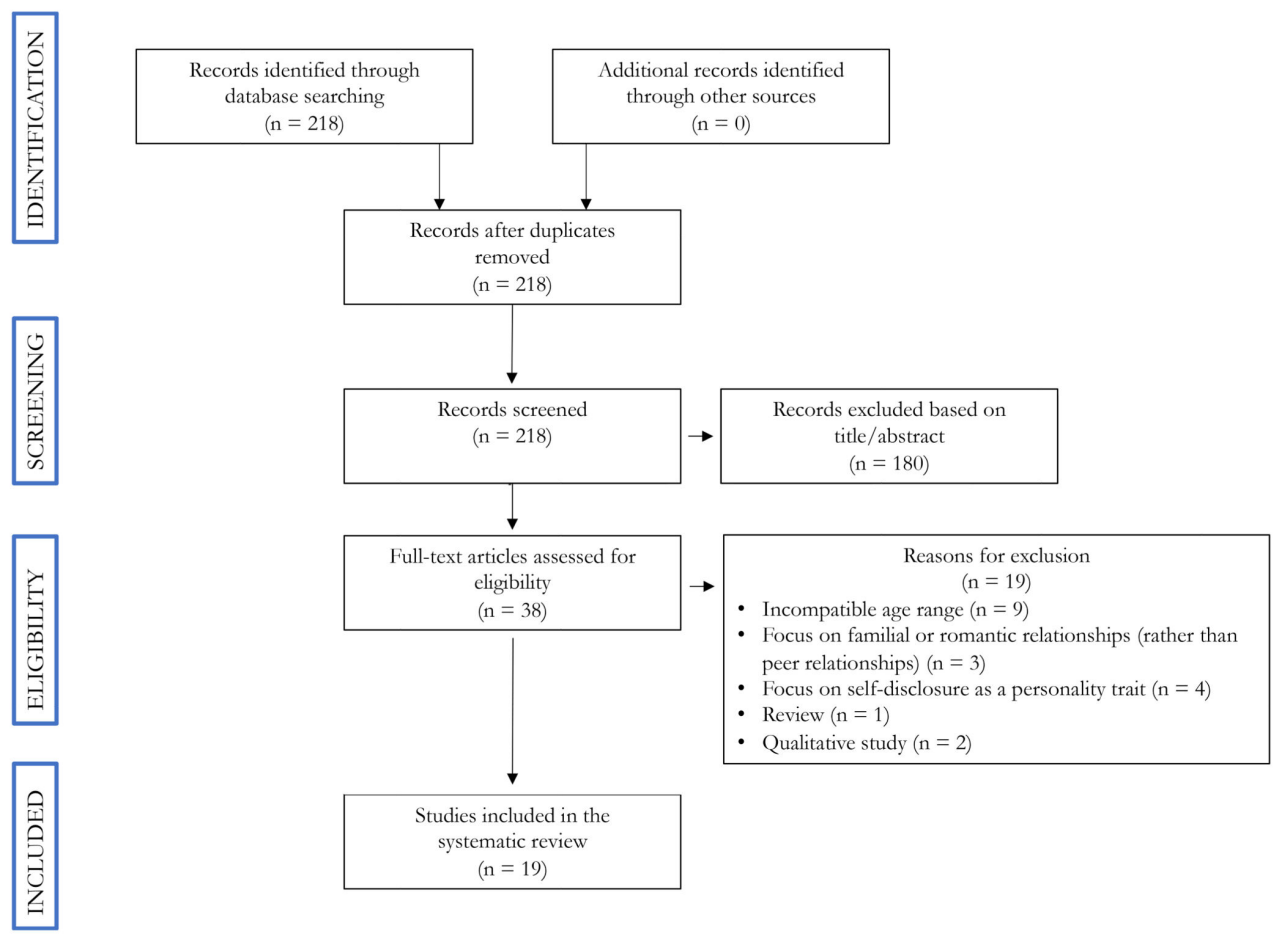
shows our search diagram in accordance with the PRISMA guidelines^21^. 218 studies were retrieved from the SCOPUS database. We determined that 38 of these studies were potentially relevant and needed to be read in full to test their eligibility. Overall, 19 studies were included in the final review based on our predetermined inclusion and exclusion criteria.

**Table 1:** A summary of the 19 studies included in the systematic review.

Source	Country of Study	Method of measuring Self-Disclosure	Sample N	Age range of Participants	Online or Offline	Summary of Main Findings	Mean Quality Assessment Score (0-100)
Schug, Yuki and Maddux (2010)	Japan and USA	A new scale was developed by combining the Self-Disclosure Scale (Miller. Berg, and Archer, 1983) and the psychological component of the Enomoto Self-Disclosure Scale (Enomoto, 1997) which could translate to both cultures.	Study 1: Japanese sample: 74 (32 female) American sample: 93 (72 female) Study 2: Japanese sample: 94 (29 female)	Study 1: Japanese Participants: M = 18.98 years, SD = 0.88 American Participants: M = 19.18 years, SD = 2.13 Study 2: M = 18.90 years, SD = 0.66	Offline	A gender difference was reported, showing that females self-disclose more than males. Even controlling for this gender difference, individuals reported engaging in higher levels of self-disclosure to strengthen their relationships with their peers.	73 (SD = 2.12)
Schiffrin, Edelman, Falkenstern, and Stewart (2010)	USA	Self-disclosure was measured via the Self-Disclosure Scale (SDS; Miller, Berg, & Archer, 1983) A modified version of the SDS was used to measure online self-disclosure.	99 (71 female)	M = 19.00 years, SD = 1.11	Both	The study showed that participants were more likely to disclose intimate information in face-to-face interactions than in computer mediated communication. Both forms of self-disclosure were positively correlated with subjective well-being	70 (SD = 0)
Valkenburg, Sumter, and Peter (2011)	The Netherlands	Self-disclosure was measured using the Online Intimate Self-Disclosure Scale (Schouten, Valkenburg and Peter’s, 2007) which is an adapted version of the Self-Disclosure Scale (Miller, Berg, & Archer, 1983)	690 (345 female)	Participants were divided into 4 age groups: -10- and 11-year-olds: N = 182, M = 10.57 years, SD = 0.56 -12- and 13-year-olds: N = 166, M = 12.54 years, SD = 0.61 -14- and 15-year-olds: N = 179, M = 14.54 years, SD = 0.55) -16- and 17–year-olds: N = 163, M = 16.51 years, SD = 0.50	Both	Gender differences were reported for adolescent self-disclosure behaviour. It was shown that both online and offline self-disclosure increased quickly between ages 10-13 years for females before plateauing, whereas this same trajectory was found later for their male counterparts (ages 12-15 years). 26 % of participants showed a preference for online self-disclosure than offline. More boys were in this group than girls – particularly boys in early adolescence (12-13 year olds) of which 40% preferred online compared to offline self-disclosure.	78 (SD = 5.66)
Courtois, All, and Vanwynsberghe (2012)	Belgium	Self-Disclosure was measured using a five-item measure obtained from Parks and Floyd (1996).	352 (246 female)	M = 16.44 years, SD = 1.39	Both	The results suggest that adolescents use computer mediated communication to learn information about their peers, something which is deemed important for establishing offline friendships, partly because it encourages self-disclosure. This was shown to be particularly important for anxious adolescents.	69 (SD = 10.61)
Huang and Yang (2013)	Taiwan	They modified Leung’s (2002) ‘Self-disclosure when chatting on ICQ’ to develop a Self-Disclosure Scale for real life and cyberspace.	608 (407 female)	Range: 13 – 18 years (no further age information provided)	Both	The authors found a negative correlation between accurate face-to-face self-disclosure and online misrepresentation. Adolescents who were more truthful in real-life Self-Disclosure engaged less in online misrepresentations.	58 (SD = 7.07)
Koutamanis, Vossen, Peter, and Valkenburg (2013)	The Netherlands	Online Self-Disclosure was measured using the Online Intimate Self-Disclosure Scale (Schouten, Valkenburg and Peter’s, 2007).	690 (345 female)	At first wave - Range: 10 – 17 years M = 13.42 years, SD = 2.27	Both	The authors found that the use of instant messaging was positively correlated with Self-Disclosure and the initiation of offline friendships.	78 (SD = 14.14)
Almquist, Ostberg, Rostila, Edling, and Rydgren (2014)	Sweden	Data was derived from a survey within the larger ‘Individual Life Chances in Social Context’ study which included measures of self-disclosure.	1289 (642 female)	All participants were born in the year 1990. Data were collected between October 2009 and January 2010.	Offline	A gender difference was found, showing that females engage in self-disclosure more than their male counterparts; while self-disclosure was only positively related to well-being in males.	84 (SD = 7.07)
Sali and Koksal Akyol (2014)	Turkey	The Peer Relationship Scale was used as a measure of self-disclosure.	1390 (534 female)	Range: 15-17 years (no further age information provided)	Offline	The authors reported a gender difference, regardless of whether the children worked or not, that females engaged more in self-disclosure than males.	59 (SD = 5.66)
Liu and Brown (2014)	China	Items from the Self-Disclosure Scale (Miller, Berg, & Archer, 1983) were adapted for the context of online communication.	264 (186 female)	M = 19.2 years (no information on SD provided)	Online	Adolescent online self-disclosure was significantly correlated with bridging social capital, an individual’s network through which they can access resources such as support, even when the individual’s social skills and gender were controlled for. Boys disclosed more demographic information (e.g., birth date, hometown, contact information, etc.) about themselves than girls.	75 (SD = 5.66)
Liu (2014)	Taiwan	A survey questionnaire with three sections was used to obtain data. The items for self-disclosure were based on work by Derlega, Metts, Petronio, and Margulis (1993);	1370 (no gender information provided)	Range: 11 – 14 years (no further information provided)	Online	The study found that as the closeness of Facebook friends’ increases, the amount of self-disclosure, intimacy, and trust increases. Additionally, the amount of self-disclosure predicted the level of intimacy with Facebook friends.	59 (SD = 5.66)
Krcmar, van der Meer, and Cingel (2015)	The Netherlands	Online self-disclosure was measured using the Online Intimate Self-Disclosure Scale (Schouten, Valkenburg and Peter’s, 2007).	381 (188 female)	M = 15.64 years, SD = 2.54	Online	Male participants reported more self-disclosure than female participants. Self-disclosure was positively associated with the personality traits narcissism and egocentrism, though the association was stronger for egocentrism. Imaginary audience ideation (i.e., the assumption that others are looking at and thinking about oneself at almost all times (Elkind 1978))is related to increased self-disclosing behaviours on Facebook.	82 (SD = 10.61)
Ang, Talib, Tan, Tan, and Yaacob (2015)	Malaysia	Online self-disclosure was measured using the Online Intimate Self-Disclosure Scale (Schouten, Valkenburg and Peter’s, 2007).	1572 (899 female)	M = 15.05 years, SD = 1.08	Online	Certain aspects of computer mediated communication (i.e., attitude toward online relationship formation) acted as predictors for online self-disclosure and the satisfaction of psychological needs by online friendships. When these needs were met, online friendships could also predict life satisfaction.	76 (SD = 12.02)
Xie and Kang (2015)	USA	Data was taken from the Teens and Privacy Management Survey, with there being 10 items specifically for measuring self-disclosure.	622 (318 female)	M = 14.94 years, SD = 1.60	Online	Self-disclosure of adolescents was shown to be positively predicted by the level of trust between them and their peers, the number of Facebook friends they had and the amount of time they spent active on social media. Boys disclosed more personal contact information than girls.	72 (SD = 14.85)
Liu, Ang and Lwin (2016a)	Singapore	A 14-item Likert scale was used to measure textual self-disclosure; a similar 8-item measure was used for visual disclosure.	780 (397 female)	M = 13.94 years, SD = 0.90	Online	The authors found a positive correlation between textual and visual adolescent self-disclosure and narcissism. The authors also report a gender difference in textual self-disclosure but not visual.	61 (SD = 7.78)
Liu, Liu, Ding, Wang, Zhen, and Xu (2016b)	China	Self-disclosure was assessed via a revised version of the Online Intimate Self-Disclosure Scale (Schouten, Valkenburg and Peter’s, 2007).	296 (175 female)	M = 16.9 years, SD = 1.36	Online	Exhibitionism, as a personality variable, was found to mediate a link between need satisfaction and online adolescent self-disclosure.	71 (SD = 4.24)
Ashktorab, Haber, Golbeck, and Vitak (2017)	USA	Self-disclosure was assessed using self-developed questions.	243 (85 female)	M = 19.6 years, SD = 0.82	Online	The results indicated that more self-disclosure leads to better relationship quality between adolescents as it also elicits greater social support.	71 (SD = 11.31)
Von Salisch (2018)	Germany	Attitudes towards self-disclosure were measured using the ‘Selbstoeffnung’. This is a 4-item self-report measure used previously by Roehrle (1994).	299 (148 female)	M = 12.6 years, SD = 0.57	Offline	Increased self-disclosure among adolescents was related to more reciprocal friendships and more meaningful disclosures over time. It was also suggested that individuals who are depressed or more prone to socially isolate themselves were less likely to disclose intimate information. Girls were shown to engage more in self-disclosure than boys.	75 (SD = 5.66)
Viktorovna, Pavlovich, and Borisovna (2019)	Russia	The authors developed a questionnaire that included items on self-disclosure behaviours in social media.	62 (26 female)	Range: 15 – 20 years (no further age information provided)	Online	The type of online self-disclosure was shown to vary with gender, with females being more likely to promote ideas or photos than males. Males also scored higher on the trait self-blame, which, like introversion, was negatively associated with online self-disclosure – adolescent males were less likely to self-disclose online than adolescent females.	51 (SD = 7.78)
Vijayakamur, Flournoy, Mills, Cheng, Mobasser, Flannery, Allen, Pfeifer (2020)	USA	Self-disclosure was measured using the Shulman’s Self Disclosure Scale (Shulman, Laursen, Kalman, Karpovsky, 1997). The value was of self-disclosure was measured as the monetary reward that participants would forgo to disclose the information.	125 (all female)	M = 11.55 years, SD = 0.81	Offline	Self-disclosure was shown as inherently rewarding as adolescents would willingly forgo monetary rewards in favour self-disclosure; it was suggested that this was because self-disclosure would help build trust and improve their relationship quality with their peers. Adolescents preferred superficial self-disclosure over intimate self-disclosure and particularly girls with high self-esteem engaged more in superficial self-disclosure.	94 (SD = 5.66)
